# Functional magnetic resonance imaging in awake transgenic fragile X rats: evidence of dysregulation in reward processing in the mesolimbic/habenular neural circuit

**DOI:** 10.1038/tp.2016.15

**Published:** 2016-03-22

**Authors:** W M Kenkel, J R Yee, K Moore, D Madularu, P Kulkarni, K Gamber, M Nedelman, C F Ferris

**Affiliations:** 1 The Kinsey Institute, Indiana University, Bloomington, IN, USA; 2 Department of Psychology, Center for Translational NeuroImaging, Northeastern University, Boston, MA, USA; 3 Brain Imaging Centre, Douglas Mental Health University Institute, McGill University, Montreal, QC, Canada; 4 SAGE Labs, St Louis, MO, USA; 5 Ekam Imaging, Boston, MA, USA

## Abstract

Anxiety and social deficits, often involving communication impairment, are fundamental clinical features of fragile X syndrome. There is growing evidence that dysregulation in reward processing is a contributing factor to the social deficits observed in many psychiatric disorders. Hence, we hypothesized that transgenic fragile X mental retardation 1 gene (*fmr1*) KO (FX) rats would display alterations in reward processing. To this end, awake control and FX rats were imaged for changes in blood oxygen level dependent (BOLD) signal intensity in response to the odor of almond, a stimulus to elicit the innate reward response. Subjects were ‘odor naive' to this evolutionarily conserved stimulus. The resulting changes in brain activity were registered to a three-dimensional segmented, annotated rat atlas delineating 171 brain regions. Both wild-type (WT) and FX rats showed robust brain activation to a rewarding almond odor, though FX rats showed an altered temporal pattern and tended to have a higher number of voxels with negative BOLD signal change from baseline. This pattern of greater negative BOLD was especially apparent in the Papez circuit, critical to emotional processing and the mesolimbic/habenular reward circuit. WT rats showed greater positive BOLD response in the supramammillary area, whereas FX rats showed greater positive BOLD response in the dorsal lateral striatum, and greater negative BOLD response in the retrosplenial cortices, the core of the accumbens and the lateral preoptic area. When tested in a freely behaving odor-investigation paradigm, FX rats failed to show the preference for almond odor which typifies WT rats. However, FX rats showed investigation profiles similar to WT when presented with social odors. These data speak to an altered processing of this highly salient novel odor in the FX phenotype and lend further support to the notion that altered reward systems in the brain may contribute to fragile X syndrome symptomology.

## Introduction

Fragile X syndrome (FXS) is the most commonly inherited cause of intellectual disability and a leading genetic cause of autism.^1, 2, 3, 4^ Indeed, nearly 30% children with FXS meet the diagnostic criteria for autism spectrum disorder (ASD). FXS is characterized by social anxiety, hyperarousal, withdrawal and gaze aversion, as well as social deficits often involving communication impairment.^5, 6, 7^ FXS is caused by a full mutation in the fragile X mental retardation 1 gene (*FMR1*). The X-linked *FMR1* gene typically contains 10–40 trinucleotide repeats of CGG. Individuals with FXS have a mutated *FMR1* gene in which the trinucleotide repeat appears over 200 times.^8, 9^ This expansion leads to hypermethylation of the promoter which silences the gene, inhibiting *fmr1* from producing fragile X mental retardation protein (FMR protein). This protein binds to mRNA and regulates synaptic communication and neural network connectivity.^10, 11^ Intriguingly, the pathophysiology of FXS can be reversed by the addition of a second mutation that restores translational homeostasis.^12^

FXS has been promoted as holding the key to understanding the pathophysiology and molecular mechanisms common to the behavioral deficits characteristic of ASD.^13, 14^ To this end, transgenic animal models of FXS have been developed to research the neurobehavioral consequences of this single gene mutation. The *fmr1*-KO mouse shows no detectable levels of *fmr1* mRNA or FMR protein and displays many of the physical and neurobiological characteristics expected of humans with FXS, for example, macroorchidism, prevalence of seizure disorders, reduced cerebellar volume and abnormalities in dendritic spines.^15, 16, 17, 18, 19^ However, the *fmr1*-tm1Cgr mouse model is not a complete molecular null model,^20^ which may contribute to discrepancies in findings. Deficits in cognitive and social behaviors in this model have been inconsistent.^18, 21^ Adult, male *fmr1*-KO mice show only mild deficits in learning.^18, 22^ Tests of social interactions, such as dominance, social novelty and social recognition are variable across studies and laboratories, and do not provide a clear behavioral phenotype.^23, 24, 25, 26^ This is also true of general anxiety. Studies under different assay conditions across many laboratories show no clear consensus.^24, 25, 27, 28, 29^ On the other hand, recent work in juvenile *fmr1-*KO mice has found a lack of social discrimination as well as behavioral hyperactivity, which may have confounded many traditional measures of sociality and anxiety.^30^ In the recently developed *fmr1*-KO rat, tests on learning, anxiety and social interaction in juveniles showed little or no difference between WT and KO animals.^31^ Olfaction appears normal in these transgenic rats as animals were able to discriminate between social and nonsocial cues. Learning and memory assessed by fear conditioning was no different as was sensorimotor gating measured by the startle response. A decrease in juvenile play was the only social deficit noted for the fmr1-KO rat. Interestingly, *fmr1*-KO rats displayed perseverative behavior as they gnawed on wood blocks in their home cage.

Do these failed efforts in transgenic rodents to present with reproducible and robust cognitive and psychosocial deficits characteristics of human FXS (that is, face validity) limit their value to the research community? Perhaps we are asking too much. No number of transgenic manipulations in a mouse or rat can recreate the range of features resembling FXS or ASD. Mental disorders are a complex interaction over time between multiple genes and the environment impacting perception, cognition and emotion that cannot be modeled in laboratory rodents.

The absence of FMR protein, a protein instrumental in development, synaptogenesis and neural connectivity is expected to have a demonstrable effect on brain function in transgenic animal models. To investigate this, we devised studies investigating changes in brain activity in awake FX and WT rats with blood oxygen level dependent (BOLD) imaging. Advances in functional magnetic resonance imaging (fMRI) in awake animals have made it possible to follow global changes in brain activity with high temporal and spatial resolution. When combined with the use of three-dimensional (3D) segmented, annotated, brain atlases and computational analysis, it is possible to reconstruct distributed, integrated neural circuits or ‘finger prints' of brain activity controlling emotional and cognitive behaviors.^32^ The present study uses odor as a provocation stimulus to activate innate evolutionary conserved sensory and motor neural circuits involved in reward—the smell of almond.^33^ Moreover, the rats used in this study were ‘odor naive'. It is only during their first imaging session that WT and FX rats are exposed to the novel stimulus of almond odor, hence the change in brain activity does not involve prior experience. We hypothesized that FX rats would show an altered response to this odor stimulus. Indeed, FX showed both positive and negative BOLD signal changes in the primary olfactory system, Papez circuit involved in emotional experience and mesolimbic/habenular reward system involved in reward-related prediction processes. These findings are discussed in the context of understanding the effects of a single gene mutation on a complex evolutionarily conserved behavior.

## Materials and methods

### Animals

Adult WT (*n*=12) and FX (*n*=10) male Sprague Dawley rats weighing ~320–350g were provided by SAGE Laboratories (St. Louis, MO, USA). The rats were maintained on a 12:12 h light:dark cycle with lights on at 0700 h. The animals were allowed access to food and water *ad libitum*. All the rats were acquired and cared for in accordance with the guidelines published in the Guide for the Care and Use of Laboratory Animals (National Institutes of Health Publications No. 85–23, Revised 1985) and adhered to the National Institutes of Health and the American Association for Laboratory Animal Science guidelines. The protocols used in this study were in compliance with the regulations of the Institutional Animal Care and Use Committee at the Northeastern University.

### Acclimation

To reduce the stress associated with head restraint, rats were acclimated to the restraining system (head holder and body tube) 1 week before their actual imaging session. The design of the restraining system (Animal Imaging Research, Holden, MA, USA) included a padded head support obviating the need for ear bars helping to reduce animal discomfort while minimizing motion artifact. These acclimation sessions were run each day for 4–5 consecutive days. The rats were briefly anesthetized with 2–3% isoflurane while being secured into the head holder. The forepaws were secured with tape. When fully conscious, the imaging system is placed into a black opaque box ‘mock scanner' for 30 min with a tape-recording of the MRI pulse sequence to simulate the bore of the magnet and the imaging protocol (Animal Imaging Research, Holden, MA USA). A significant decline in respiration, heart rate, motor movements and plasma corticosterone has been measured when the first and last acclimation periods are compared.^34^ This reduction in autonomic and somatic measures of arousal and stress improve the signal resolution and quality of the images. If subjects exceed motion in any dimension equal to or greater than a single voxel's width (312 μm), their data are excluded from the analysis. No such exclusions were performed with these data.

### Image acquisition

The animals were scanned at 300 MHz using a quadrature transmit/receive volume coil built into the rat head holder and restraining system for awake animal imaging (Animal Imaging Research). A video of the rat preparation for imaging is available at www.youtube.com/watch?v=JQX1wgOV3K4. The design of the coil provided complete coverage of the brain from olfactory bulbs to brain stem with excellent B1 field homogeneity. Experiments were conducted using a Bruker Biospec 7.0T/20-cm USR horizontal magnet (Bruker, Billerica, MA, USA) and a 20-G/cm magnetic field gradient insert (ID=12 cm) capable of a 120-μs rise time. At the beginning of each imaging session, a high-resolution anatomical data set was collected using the RARE pulse sequence (22 slice; 1.0 mm; field of vision=3.0 cm; 256 × 256; repetition time=2.5 s; echo time=12 ms; NEX 2; 2-min acquisition time). Functional images were acquired using a multislice HASTE pulse sequence (Half Fourier Acquisition Single Shot Turbo Spin Echo). Bruker Paravision automatically finds the basic frequency, shims, power requirements for 90^o^ and 180^o^ pulses and sets the receiver gain. A single scanning session acquired 22 slices, 1.0 mm thick, every 6.0 s (repetition time=6.0 s, echo time=48 ms, field of vision=3.0 cm, matrix size 96 × 96, NEX 1) repeated 90 times for a total time of 9 min. The in-plane pixel resolution is 312 μm^2^. Each scanning session was continuous, starting with 40 baseline image acquisitions, followed by odor presentation over the next 30 image acquisitions, cessation of odor and a final 20-image acquisitions.

**Figure 1:**
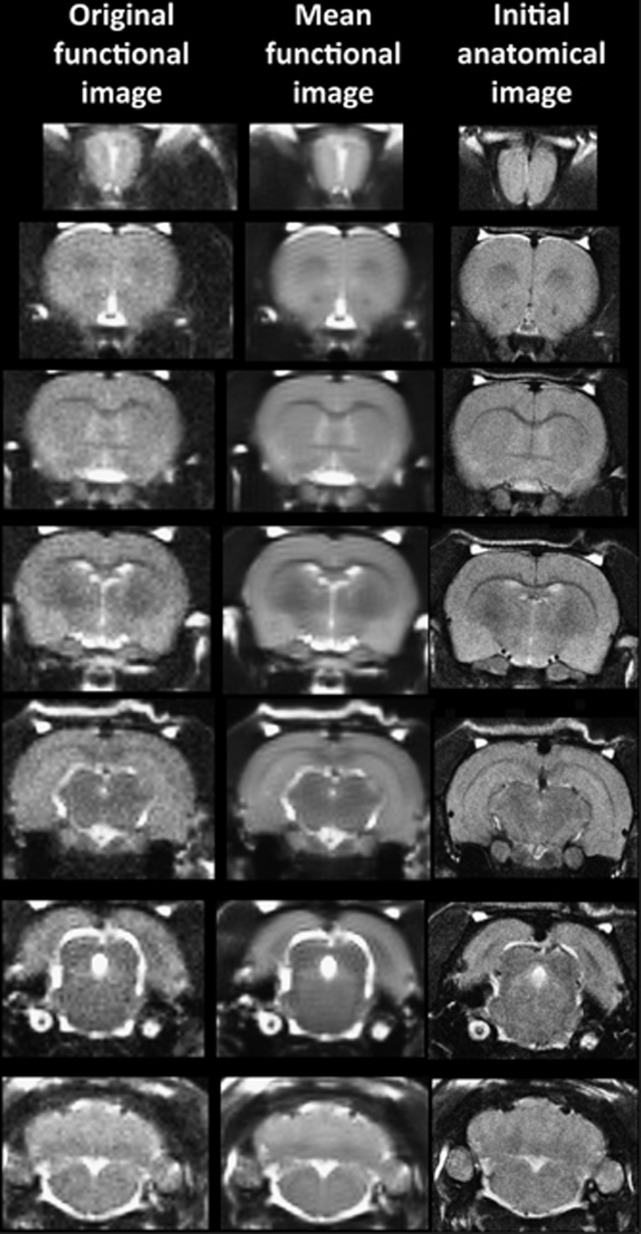
Neuroanatomical fidelity shown are representative examples of brain images collected during a single imaging session using a multislice spin echo, RARE (rapid acquisition with relaxation enhancement) pulse sequence. The column on the right shows axial sections collected during the anatomical scan taken at the beginning of each imaging session using a data matrix of 256 × 256, 22 slices in a field of view of 3.0 cm. The column on the left shows the same images but collected for functional analysis using HASTE, a RARE pulse sequence modified for faster acquisition time. These images were acquired using the same field of view and slice anatomy but a larger data matrix of 96 × 96. The images in the middle column have been smoothed during pre-processing. Note the anatomical fidelity between the functional images and their original anatomical image. The absence of any distortion is necessary when registering the data to atlas to resolve 171 segmented brain areas. HASTE, Half Fourier Acquisition Single Shot Turbo Spin Echo.

It should also be emphasized that high neuroanatomical fidelity and spatial resolution are critical in identifying distributed neural circuits in any animal imaging study. Many brain areas in a segmented rat atlas have in-plane boundaries of less than 400 μm^2^ and may extend for over 1000 μm in the rostral/caudal plane. With the development of a segmented, annotated 3D MRI atlas for rats (Ekam Solutions, Boston, MA, USA), it is now possible to localize functional imaging data to precise 3D ‘volumes of interest' in clearly delineated brain areas. Therefore, it is critical that the functional images are a very accurate reconstruction of the original brain neuroanatomy as shown in Figure 1.

The HASTE sequence, a spin echo multislice pulse sequence used in these studies, corrects for field inhomogeneity, susceptibility artifact, chemical shift and other imaging distortions and does not require any additional shimming as would be the case for gradient echo pulse sequences that are commonly used in BOLD imaging studies. The major disadvantage to the HASTE sequence as compared with gradient echo is the loss of signal contrast. The problem of sensitivity can be addressed with higher field strengths as used here (7T) where the BOLD signal becomes a function of dynamic dephasing from diffusion of water at the level of the capillaries.^35, 36^ Using multislice, fast-spin echo sequences, the signal contrast with BOLD imaging is a function of T2 and not T2* at high-field strengths. The extravascular signal surrounding capillary beds and small vessels is more reflective of the metabolic changes in brain parenchyma than signal from large draining veins helping to improve the localization of the signal changes.^37^ The BOLD signal is linear and reproducible at stimulus intervals of 1 s.^38^

### Data analysis

The data are coregistered to a mean functional image using the coregistrational code of SPM8 with the following parameters: quality: 0.97, smoothing: 0.35 mm, separation: 0.5 mm. Gaussian smoothing was performed with a full width at half maximum of 0.8 mm. The preprocessed functional files were then exported to Medical Image Visualization and Analysis for registration and segmentation. Images were aligned and registered to a 3D rat brain atlas, which is segmented and labeled with 171 discrete anatomical regions. The alignment process was facilitated by an interactive graphic user interface. The registration process involved translation, rotation and scaling independently and in all three dimensions. Matrices that transformed each subject's anatomy were used to embed each slice within the atlas. All pixel locations of anatomy that were transformed were tagged with regions of interest (ROIs) in the atlas. This combination created a fully segmented representation of each subject within the atlas. The alignment was carried out by trained researchers blind to the experimental group.

In voxel-based analysis, the BOLD % change of each independent voxel was averaged for all the subjects with a baseline threshold of 2% BOLD change to account for normal fluctuation of BOLD signal in the rat brain.^39^ A composite image of the whole brain representing the average of all the subjects was constructed for each group for ROI analyses, allowing us to look at each ROI separately to determine the BOLD change and the number of activated voxels in each ROI. The statistical *t*-tests were performed on each voxel (~15 000 in number) of each subject within their original coordinate system. The average signal intensity in each voxel of minutes 2–4 of baseline (acquisitions 11–40) was compared with minutes 4–7 (acquisitions 41–70). The *t*-test statistics used a 95% confidence level, two-tailed distributions and heteroscedastic variance assumptions. As a result of the multiple *t*-test analyses performed, a false-positive detection controlling mechanism was introduced.^40^ This subsequent filter guaranteed that, on average, the false-positive detection rate was below our cutoff of 0.05.

The volume of activation was compared across the experimental groups using the nonparametric Kruskall–Wallis test statistic followed by *post hoc* analyses using a Wilcoxon rank-sum test for individual differences. The brain areas were considered statistically different between experimental groups when comparison produced *P*-values less than or equal to our cutoff of 0.05. The sample sizes were chosen on the basis of estimated effect sizes derived from previous experience with awake animal fMRI.

### Provocation paradigm—odor of almond

The awake WT and FX rats were imaged for changes in BOLD signal intensity in response to the odor of almond (benzaldehyde), a stimulus to elicit the innate reward response.^33^ All the animals were ‘odor naive' to these evolutionarily conserved stimuli raising the following question. How has this single gene mutation altered the perception of this conserved odorant signal important for survival? We chose the almond odor because nuts are high in calories and would be expected to convey greater valance as compared with the other odors. Moreover, the standard food chow is devoid of nuts, so laboratory-bred rats and the rats used in these studies have no previous exposure to this food. In a recent study,^33^ brain-activation maps from the odors of banana, rose, citrus and almond were dramatically different. Almond, but not the other odors, activated the hippocampus, amygdala and limbic cortex. In a serial dilution study for almond scent, we identified a threshold dilution of 100% benzaldehyde (1/10 000 v/v) that gives a significant and consistent pattern of brain activity. This threshold dilution of almond odor was used in this study. Subjects were also imaged for BOLD signal change in response to ambient room air (AIR condition). The testing for almond and AIR occurred in randomized order on separate days, 48 h apart.

### Normalization of volume of activation

The differences BOLD signal change in wild-type and FX rats are reported in terms of volume of activation or number of voxels per ROI or brain area. In this study, the brain size across phenotypes can be significantly different (see Supplementary Table 2) from each other hence the volume of activation is normalized to volume of ROI. By normalizing to volume of activation, we can compare across different phenotypes or within group among different regions. The normalized volume of activation was computed using the following formula:


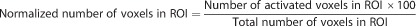


### Calculating the volumes of different brain areas

The volume of each brain area (ROI) was determined from the high-resolution anatomical scan taken at the beginning of each scanning session for each subject. The 3D segmented atlas provides the precise number of voxels (3D pixels) that combine to fill the volume of each of the 171 ROIs or brain regions. The dimensions of each voxel are calculated from the slice thickness (0.75 mm), voxel width (field of vision in *x* direction/number of voxels in *x* direction) and voxel height (field of vision in *y* direction/number of voxels in *y* direction) using the formula:


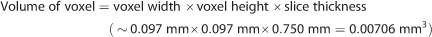


The total number of voxels in each ROI was multiplied by the volume of voxel to compute the total volume of the brain region.

### Behavioral tests

The WT and FX rats were tested for evidence of nonsocial odor preference in a novel chamber equipped with three odors as well as saline vehicle. The testing chamber consisted of an enclosure (60 × 70 × 70 cm). Each corner contained a perforated tube approximately 25 cm above the floor of the environment. Each tube contained two small Kimwipe sheets soaked with odorant consisting of 1 ml of a 1% solution (odorant/saline) of benzaldehyde (almond odor), iso-amyl acetate (banana odor), methyl benzoate (rose odor) or saline vehicle. The rats were introduced individually into the corner containing the saline stimulus and remained in the testing chamber to freely explore for 10 min. The testing was done 5–10 days following the fMRI scans, thus while the rats were naive to the banana and rosy odors, they had been previously exposed to almond under potentially aversive conditions. The behavior was recorded from overhead and scored later by two trained, experimentally blind observers using Noldus Observer (Noldus, Wageningen, The Netherlands). The time spent sniffing was quantified and defined as rearing on the hind legs with the nose within 2 cm of the tube.

In a subsequent experiment, the WT and FX rats were tested for evidence of odor preference in the same chamber equipped with three social odors as well as saline vehicle. Each tube contained the odors of WT rats in two shredded Kimwipe sheets in one of the following three conditions: (1) male, (2) female, metestrus/diestrus or (3) female, estrus; a fourth tube contained saline. For the ‘male' tube, Kimwipe sheets contained only urine from a set of male WT Sprague Dawley rats. The two ‘female' tubes contained a mixture of urine and vaginal lavage (performed with saline) from a set of female WT Sprague Dawley rats that were identified as either metestrus/diestrus or estrus through inspection of vaginal cytology. The subjects were introduced individually into the corner containing the saline stimulus and remained in the testing chamber to freely explore for 10 min. The testing occurred 10–15 days following the fMRI scans. The behavior was recorded from overhead and scored later by two trained, experimentally blind observers using Noldus Observer (Noldus). The time spent sniffing was quantified and defined as rearing on the hind legs with the nose within 2 cm of the tube.

## Results

### fMRI scan

**Figure 2:**
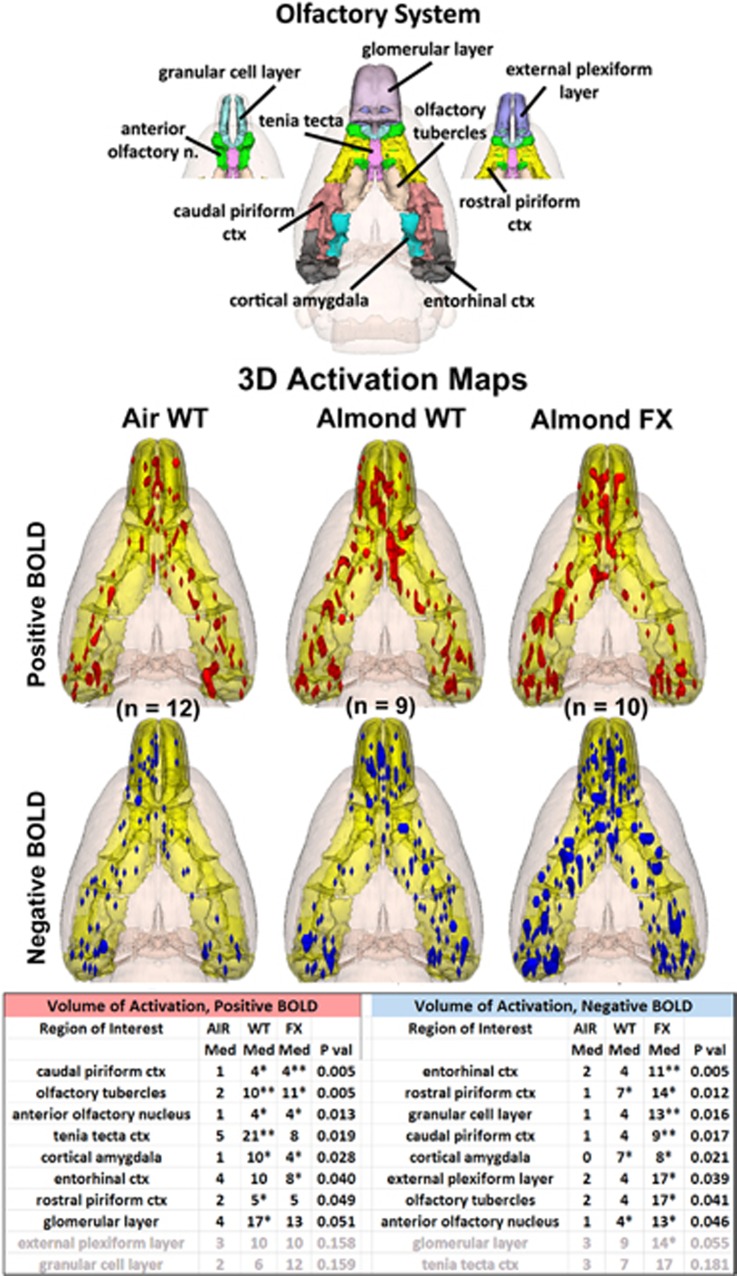
Primary olfactory system shown above are three-dimensional (3D) colored volumes of 10 areas comprising the primary olfactory system. The color-coded volumes are coalesced into a single volume shown in yellow below for each of the three experimental conditions: ambient air in wild-type Sprague Dawley controls (Air WT), almond odor in WT and almond odor in fragile X KO rats (FX). The number of animals contributing to the data for each experimental condition are shown in parentheses. Once fully registered and segmented, the statistical responses for each animal are averaged on a voxel-by-voxel basis. Those averaged voxels that are significantly different from baseline for positive and negative BOLD are shown in their appropriate spatial location coalesced as a 3D volume. Below are tables of these regions of interest for negative and positive BOLD. The columns show the median (Med) number of significant voxels for each brain area for each experimental condition. The voxel numbers for each condition were analyzed using a Newman–Keuls multiple comparisons test statistic followed by *post hoc* analyses using a Wilcoxon rank-sum test for individual differences. All the areas are ranked in the order of their significance. There were no differences between WT and FX for positive BOLD. Only the olfactory tubercles showed a significant difference between these two groups (*P*<0.05) for negative BOLD. All the other differences are voxel numbers greater than AIR. *P<0.05, **P<0.01. BOLD, blood oxygen level dependent.

The 3D color model at the top of Figure 2 shows the different areas comprising the primary olfactory system. These areas have been coalesced into a single volume (yellow) as shown in the lower 3D images for each of the three experimental conditions. The areas in red and blue are the composite average of the significant increase in volumes of activation (number of voxels in a ROI) for positive BOLD and negative BOLD, respectively. The median (Med) number of positive and negative voxels activated for each experimental condition are shown in the Supplementary Tables. These brain areas are ranked in the order of their significance. These data from all the brain areas are presented in the 3D activation maps above. For a complete summary of all the 171 brain areas, positive and negative BOLD, see Supplementary Tables 1 and 2.

The 3D activation maps present patterns of positive BOLD signal that show greater signal change in WT and FX in response to almond odor as compared with AIR control, as shown in Figure 2. This difference in positive BOLD is shown in Supplementary Table 1. Both WT and FX rats showed significant increases in positive BOLD volume of activation in several areas of the primary olfactory system as compared with ambient air (AIR). Relative to baseline, WT showed significant activation in 7/10 brain regions: rostral piriform cortex, caudal piriform cortex, olfactory tubercles, anterior olfactory nucleus, tenia tecta, cortical amygdala and the glomerular layer of the olfactory. FX rats showed significant activation in 5/10: caudal piriform cortex, olfactory tubercles, anterior olfactory nucleus, cortical amygdala and entorhinal cortex. There were no significant differences between WT and FX for positive BOLD. FX animals showed significantly higher negative BOLD volumes of activation across 9/10 areas of the primary olfactory system (all but tenia tecta) relative to baseline, whereas WT only showed three: the rostral piriform cortex, cortical amygdala and anterior olfactory nucleus, though there were no significant differences in negative BOLD volume of activation between WT and FX.

**Figure 3:**
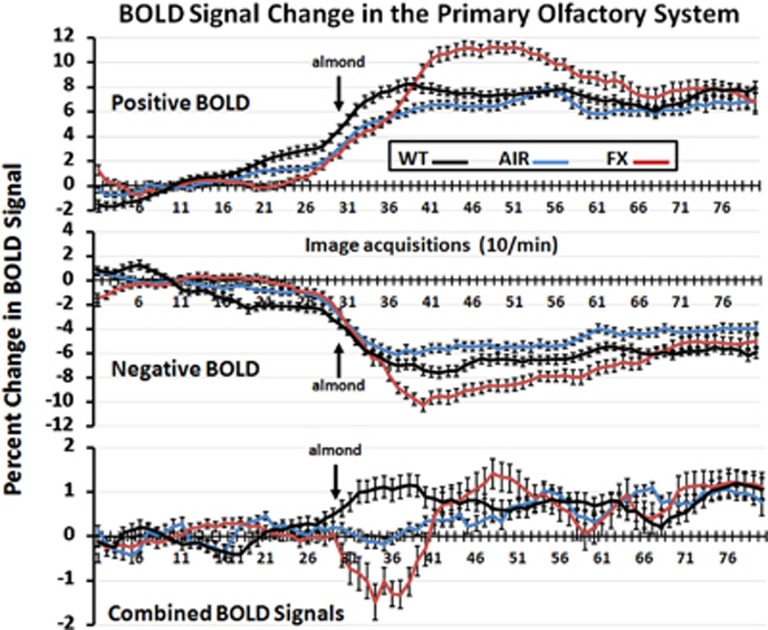
Time course data for BOLD signal change in primary olfactory system shown are the changes in the positive and negative BOLD signal and the combined signal over time in the primary olfactory system following exposure to almond (arrow) for wild-type (WT) and fragile X knockout (FX KO) or ambient air to AIR. Vertical bars denote s.e.m. BOLD, blood oxygen level dependent.

The pattern of BOLD signal change over time in response to almond odor for WT and FX and ambient air for AIR controls is shown in Figure 3. These data combine all the 10 brain areas that comprise the primary olfactory system (see Figure 2). The percent change in positive BOLD, negative BOLD and both BOLD signals combined are significantly different over time (positive BOLD: F_2,158_=9.19, *P*<0.0001; negative BOLD: F_2,158_=8.49, *P*<0.0001; combined BOLD: F_2,158_=5.20, *P*<0.0001). Although there was no difference in the AIR controls (combined BOLD: *t*_119_=−0.138, *P*=0.89), each experimental condition is significantly different from its 3-min baseline (combined BOLD_WT-BENZ_: *t*_89_=−5.13, *P*<0.0001; combined BOLD_FX-BENZ_: *t*_99_=3.57, *P*=0.0006) and from each other in the first 10 min of the odor stimulus period (WT-AIR vs WT-BENZ: *μ*_diff_=−0.945, *P*=0.0009; WT-BENZ vs FX-BENZ: *μ*_diff_=−1.90, *P<*0.0001; WT-AIR vs FX-BENZ: *μ*_diff_=−0.954, *P*=0.0005).

**Figure 4:**
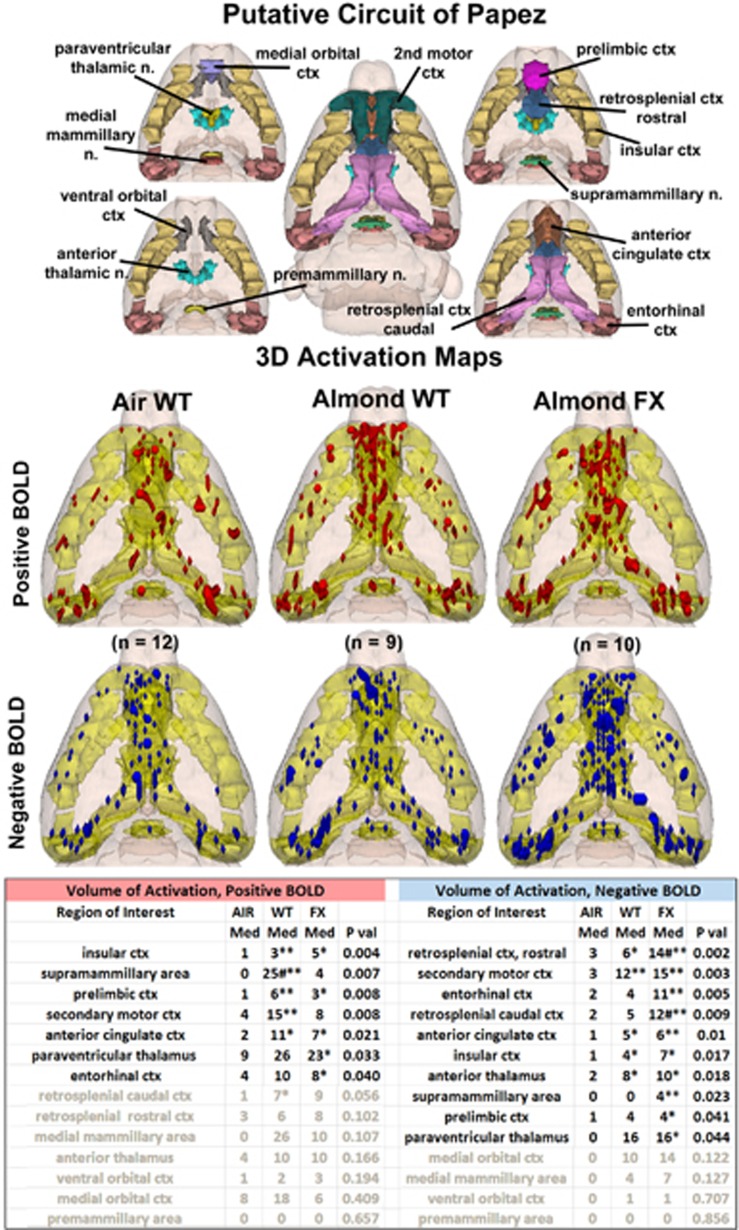
Papez circuit of emotional experience shown are three-dimensional (3D) renderings of 14 volumes comprising the putative neural circuit of Papez involved in emotional experience. This circuit was taken from Kulkarni *et al.*^33^ The color-coded volumes are coalesced into a single volume shown in yellow. *P<0.05 compared to AIR; **P<0.01 compared to AIR; ^#^*P*<0.05 compared between WT and FX. The description of data presentation is the same as Figure 2.

Shown in Figure 4 are the patterns of BOLD signal change for each experimental condition in the putative circuit of Papez, a thalamic, limbic cortical network associated with emotional experience. The 3D activation maps present patterns of positive BOLD signal that show greater signal change in WT and FX in response to almond as compared with AIR control. This difference in positive BOLD is shown in Supplementary Table 1. Both WT and FX rats showed significant positive BOLD volume of activation when exposed to the odor of almond as compared with ambient air. Relative to the baseline, WT showed activation in 6/14 areas (insular cortex, supramammillary area, prelimbic cortex, secondary motor cortex, anterior cingulate cortex and caudal retrosplenial cortex), while FX showed positive activation in 5/14 areas (insular cortex, prelimbic cortex, anterior cingulate cortex, paraventricular nucleus of the thalamus and entorhinal cortex). The supramammillary area in WT was significantly higher than in FX. Other than this brain area, there were no differences in positive BOLD between WT and FX in response to almond odor. WT showed significant negative BOLD in 5/14 brain areas, while FX showed significant changes in 10/14 areas as compared with AIR (FX: rostral retrosplenial cortex, caudal retrosplenial cortex, secondary motor cortex, entorhinal cortex, anterior cingulate cortex, insular cortex, anterior thalamus, supramammillary area, prelimbic cortex and paraventricular nucleus of the thalamus; WT: rostral retrosplenial cortex, secondary motor cortex, anterior cingulate cortex, insular cortex and anterior thalamus). In two areas, the retrosplenial cortices, negative BOLD volume of activation in FX rats was significantly greater than WT. There was a trend toward greater negative BOLD in FX rats across areas of Papez circuit when compared with WT.

**Figure 5:**
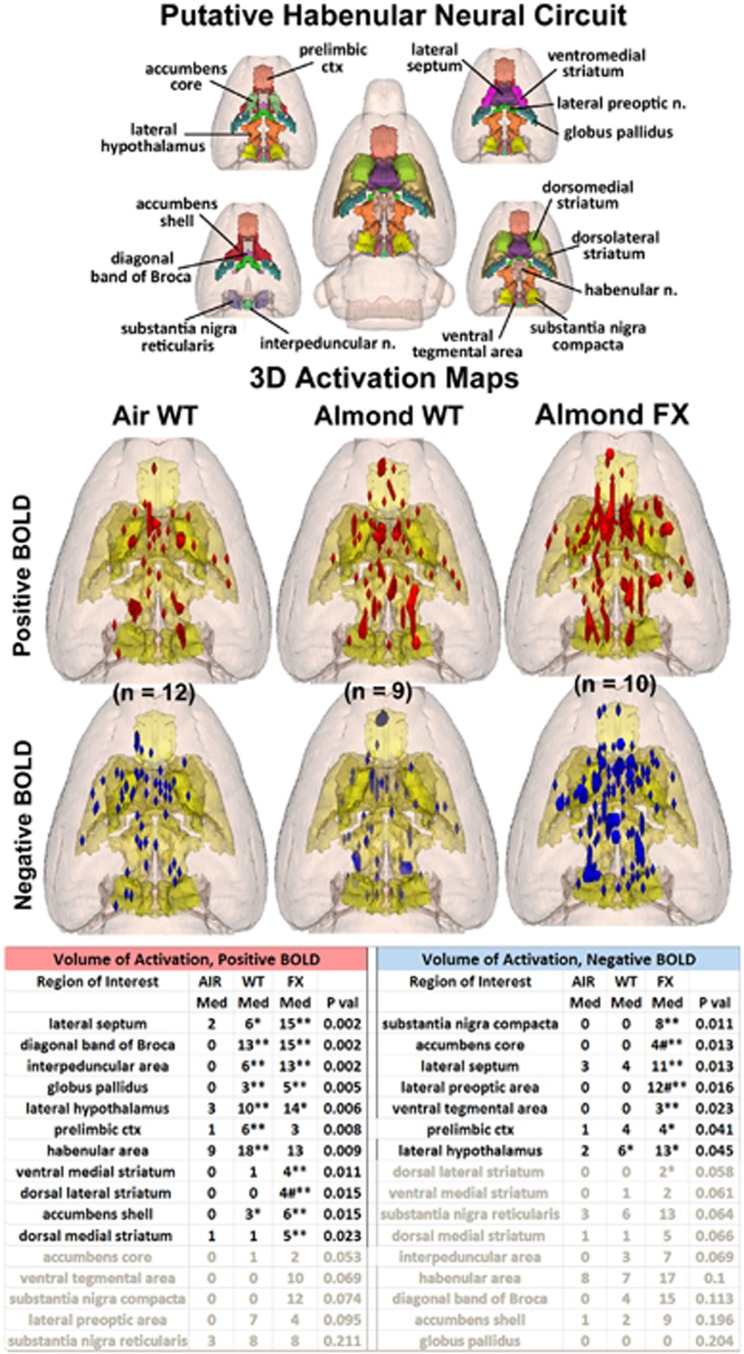
Putative habenular neural circuit shown above are three-dimensional (3D) colored volumes of 16 areas comprising the putative habenular neural circuit. The color-coded volumes are coalesced into a single volume shown in yellow below for each of the three experimental conditions. The description of data presentation is the same as Figure 2. There were no differences between WT and FX for positive BOLD except the dorsal medial striatum (^#^*P*<0.05). Several areas were different between WT and FX for negative BOLD. All the other differences are voxel numbers greater than AIR. *P<0.05, **P<0.01. BOLD, blood oxygen level dependent; FX, fragile X; WT, wild type.

In the mesolimbic/habenular reward neural circuit, as shown in Figure 5, the differences between WT and FX animals in response to almond are displayed in the positive and negative BOLD activation maps. In this neural circuit, associated with reward and the prediction of adverse negative events, there was a significant difference between WT and FX rats as shown in the Supplementary Tables. As compared with AIR, WT rats showed positive volume of activation in 8/16 brain areas, while FX rats showed significant activation as compared with ambient air in 9/16 brain areas (FX: lateral septum, diagonal band of broca, interpeduncular area, globus pallidus, lateral hypothalamus, prelimbic cortex and accumbens shell; WT: lateral septum, diagonal band of Broca, interpeduncular area, globus pallidus, lateral hypothalamus, ventral medial striatum, dorsal lateral striatum and accumbens shell). The dorsal lateral striatum showed significantly greater positive BOLD in the FX rats compared with WT. The pattern across most of the mesolimbic/habenular system for volume of positive activation was greatest in FX as seen in the 3D activation maps, with the exception of prelimbic cortex and the habenula, which showed greater activation in WT than FX. The pattern of negative BOLD volume of activation showed greater differentiation as WT presented with only one significant difference compared with ambient air (lateral hypothalamus), whereas FX rats showed significance in 8/16 brain areas: substantia nigra pars compacta, accumbens core, lateral septum, lateral preoptic area, ventral tegmental area, prelimbic cortex, lateral hypothalamus and dorsal lateral striatum. Two of these areas, accumbens core and lateral preoptic area were also significantly different between FX and WT, with FX showing greater negative BOLD.

### Odor discrimination

**Figure 6:**
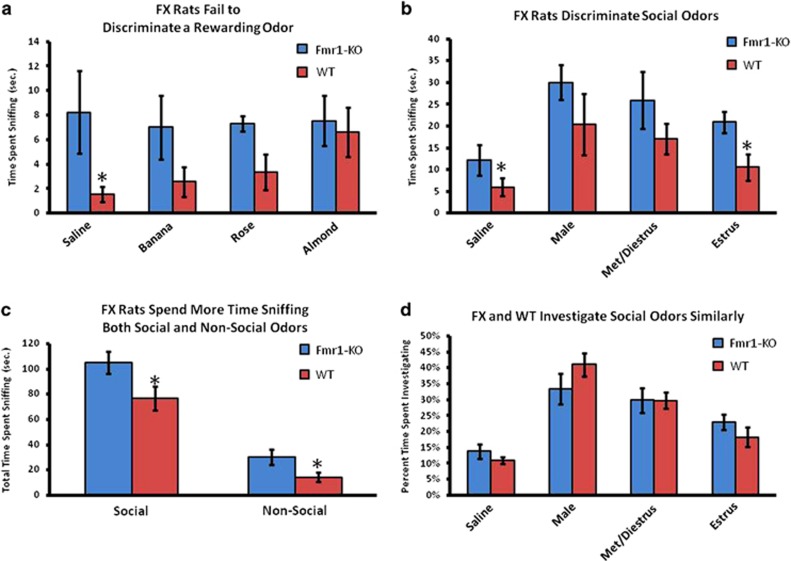
Olfactory discrimination shown are the times spent investigating various odors for WT and FX rats in freely behaving preference tests. All the odors besides almond were novel to the rats. WT rats showed a preference for almond, as evidenced by a significantly greater time spent exploring that odor relative to saline (**a**, *P<0.05), whereas FX rats showed no clear preference. FX animals spent more time sniffing saline and estrus female odor compared with WT (**b**, *P<0.05), though when this is expressed as a proportion of total time spent sniffing, there were no differences between FX and WT (**d**). FX spent a greater time overall investigating all the odors (**c**, *P<0.05). FX, fragile X; WT, wild type.

The WT animals spent more time investigating almond odor relative to saline vehicle (*P*<0.05), whereas the FX animals showed no such preference (Figure 6). Indeed, the FX animals displayed high levels of sniffing toward all nonsocial odorants and thus spent a greater total time sniffing nonsocial odor sources as compared with WT (*P*<0.05). Similarly, the FX animals displayed higher levels of sniffing all social odorants and thus spent a greater total time sniffing social odor sources as well (*P*<0.05). Both the WT and FX rats spent more time investigating social odors relative to saline vehicle (*P*<0.05). FX rats spent more time investigating the saline and estrus female odors (*P*<0.05), however, when the data were transformed to evaluate time spent investigating each social odor as a proportion of total time spent investigating all social odors, there were no differences between FX and WT, which shows that the groups' overall investigation profiles were broadly similar.

## Discussion

The present studies were undertaken to determine whether there were differences in brain activity between the WT and FX rats given the evidence from many studies showing little, if any, differences in behavioral measures of social behavior, learning and memory in rodents.^18, 31^ We assumed that this single gene mutation, germane for brain development, must have some impact on neural processing associated with environmental stimuli that is not discernible in behavioral models. To test this, we used BOLD imaging in awake rats exposed to the odor of benzaldehyde, otherwise recognized as the smell of almond. We chose almond as the odor stimulus because rats have genes that code for the benzaldehyde-sensitive odorant receptor M71 localized to olfactory sensory neurons.^41^ The stimulation of these receptors in the olfactory epithelium with the odor of benzaldehyde produces activation maps onto specific areas of the glomerular layer of the olfactory bulb.^42^ In an earlier study, Kulkarni *et al.*^33^ showed that ‘odor naive' rats exposed to almond odor for the first time showed activation across multiple neural circuits involved in emotion and memory. The results from WT subjects of the present study largely agree with the pattern of activation seen in response to almond in this earlier work. This suggests that the ‘odorant code' for benzaldehyde extends beyond the olfactory bulb to include hardwired neural networks conserved over evolution to reinforce adaptive behavior critical for survival. The rats used in the present fMRI experiment were also ‘odor naive', that is, never exposed to the smell of almond. This is important for two reasons: (1) the first exposure elicits brain activity in evolutionarily conserved neural circuits associated with reward processing and (2) the change in brain activity is independent of learning. This sets the framework for the discussion to follow, and the compelling question—how has a single gene mutation in FX rats affected their ability to process a highly rewarding, evolutionarily conserved stimulus as compared with WT controls?

From the 3D images of positive BOLD in Figure 2, there is no discernible difference between the WT and FX rats. Both show activation of the primary olfactory system that is significantly greater than control animals exposed to a stream of ambient air over their nose. The pattern of significant positive BOLD activity in the primary olfactory system included more brain areas in the WT than FX, and favored greater voxel numbers in the WT over FX, but the latter was not significantly different. The most notable difference between WT and FX is the degree of negative BOLD volume of activation, which is shown in the 3D images in Figure 2. All the brain areas that comprise the primary olfactory system, except one, presented with significantly more negative BOLD voxels in FX as compared with AIR. In contrast, there was very little difference between AIR and WT for negative BOLD volume of activation. This pattern of BOLD signal change in the olfactory system suggests similar activation in WT and FX in response to almond odor, but a simultaneous reduction in activity in FX as reflected by the negative BOLD. This negative/positive profile of BOLD signal change can also be seen in Figure 3 showing the change in BOLD signal over time. The FX rats exposed to almond presented with positive and negative changes in BOLD signal in the primary olfactory system that significantly exceeded WT. Although these BOLD signals come from different voxels located in the same brain areas, they can be combined yielding an average BOLD signal change for any brain area. Interestingly, the average of the negative and positive BOLD for WT and FX shows a transient net negative BOLD for FX and positive BOLD for WT lasting for approximately 1 min over the primary olfactory system. The behavioral studies on odor detection (see Figure 6) show that the FX rats can detect odors but that there is no clear discrimination as compared with the WT. Perhaps the inability to discriminate and attend to the valence of an odor stimulus is somehow attributed to the high level of negative BOLD over much of the olfactory system. Although FX rats did not discriminate between saline and nonsocial odors, they did, however, spend more time investigating social odors in comparison with saline, demonstrating at least partial olfactory discrimination. Interestingly, FX rats showed a similar investigation profile to social odors as did WT rats, though again, they spent more time overall sniffing all the social odorants. Thus, we are left with a FX phenotype marked by (1) a lack of behavioral preference for a hedonically rewarding, nonsocial stimulus; (2) an investigation profile of social stimuli similar to WT; and (3) an increase in time spent investigating both social and nonsocial stimuli. We hypothesize that the first finding (together with the BOLD results of this study) suggest alterations to reward processing in the FX rats. The second and third findings together suggest the possibility that increased investigation time may translate into alterations in species-typical social behavior. Finally, the third finding may be explained by a number of different underlying mechanisms such as increased salience of all olfactory information, decreased working memory and/or decreased neophobia/anxiety.

Processing the ‘emotional experience' of any environmental stimulus involves a constellation of brain areas particularly limbic cortex, hippocampus and amygdala. Many of the brain areas comprising these neural circuits are activated in both WT and FX in response to almond odor (see Supplementary Table 1). As an example of limbic cortex, Figure 4 shows the putative neural circuit of Papez.^43^ The ‘Papez circuit' connects the hypothalamus and hippocampus to the limbic cortex through the dorsal midline thalamus (anterior and posterior thalamic areas). The pattern of positive and negative BOLD presented by the WT and FX rats in response to almond is similar to that of the primary olfactory system. Although both the experimental groups show several brain areas that are significantly greater than AIR, there is no ostensible difference in the Papez circuit between the WT and FX rats as seen in the 3D positive BOLD activation maps. Again the trend across most brain areas favors greater positive voxel numbers for WT vs FX and greater negative voxel numbers for FX vs WT. Although there were only a few differences in negative BOLD between WT and AIR, the pronounced negative BOLD in FX vs WT and AIR is seen in the 3D activation maps. Does this negative BOLD over much of Papez circuit in FX affect their ability to synthesize information about an emotional and cognitive experience?

While reviewing the brain areas that were affected by almond in WT and FX (see Supplementary Table 1), it was noted that many of the areas comprising the mesolimbic/habenular reward circuit were activated. This circuit includes the areas classically associated with reward, namely the nucleus accumbens, ventral tegmental area and striatum. The first awake animal imaging template of the mesolimbic/habenular neural circuit to focus on the habenula was recently published by Yee *et al.*^44^ in a study on pain. Activation of the habenuIar system was interpreted in the context of predicting aversive events.^45, 46^ However, more often, the habenula is viewed in light of its role in reward processing and motivational control of behavior related to reward prediction error (the actual reward value vs the expected reward value).^47, 48^ Hence, it is not surprising that this neural circuit is activated by an evolutionarily conserved, hedonic stimulus. The habenula is part of an integrated neural circuit comprising the basal ganglia, midbrain dopaminergic neurons, hypothalamus and forebrain. Subdivisions of these reward-associated regions showed greater positive BOLD signal in FX animals; the dorsal lateral striatum showed significantly greater positive in BOLD signal in FX, while the accumbens core showed greater negative BOLD signal. Interestingly, the habenula and prelimbic cortex were the only two regions in this circuit that showed higher positive BOLD signal in WT as compared with FX animals. Activation of the habenula inhibits substantia nigra compacta and ventral tegmental area and reduces dopaminergic neurotransmission.^49^ In the context of predicting reward, the habenula is highly activated when reward is not forthcoming and not activated when reward is present.^50^ The habenula is also known to process olfactory responses, especially under stressful/aversive conditions.^51^

The 3D activation maps in Figure 5 illustrate greater positive and negative BOLD signal change in FX compared with WT in response to almond odor. Both WT and FX show many areas in the mesolimbic/habenular neural circuit that are significantly different than AIR. Interestingly, the habenula shows high positive volume of activation in all the three experimental conditions. Unlike the previous neural circuits, the trend toward greater numbers of positive voxels favors FX, (except in the habenula and prelimbic cortex). Meanwhile, the pattern of negative BOLD signal change remains the same, as the FX show a significant increase in negative voxels in the substantia nigra compacta, ventral tegmental area, accumbens and several other areas while the change in negative BOLD is negligible for WT. Indeed, there is no difference between WT and AIR in negative BOLD other than the lateral hypothalamus. From these data, we can conclude that the mesolimbic/habenular reward circuit in FX is very sensitive to almond odor, resulting in a pattern of BOLD signal change that suggests an alteration of dopamine neurotransmission not seen in WT.

Here we report functional and anatomical differences in the dorsal lateral striatum, accumbens core and lateral preoptic area in FX. These are among the first data connecting the mesolimbic/habenular neural circuit with FXS and raises the possibility that the social deficits observed in FXS and ADS may be due to problems associated with reward processing and its known regulators, including dopamine. Indeed, the link between FXS and altered dopamine signaling was made by Wang *et al.*^52^ reporting cultured neurons from the prefrontal cortex (what would be prelimbic cortex in this study) and striatum from *fmr1*-KO mice showed impaired dopamine D1 receptor transduction. The absence of FMR protein leads to deregulation of G protein-coupled receptor kinase 2 and the hypothesized impairment of D1 receptor-mediated signaling in the forebrain. In a later study, Fulks *et al.*^53^ using brain slices from the striatum of *fmr1*-KO mice reported impaired release and uptake of dopamine. Dopaminergic modulation of synaptic transmission also appears disrupted in the prefrontal cortex in *fmr1*-KO mice.^54^ Indeed, evidence has been mounting for dysregulated dopamine signaling in FXS.^55, 56^ Most recently, the accumbens has been found to be an area of disrupted long-term potentiation and disrupted synaptic functioning in a mouse model of FXS^57^ and in the same study that observed hyperactivity and a lack of social discrimination, an increase in striatal dopamine was also observed in *fmr1*-KO mice.^30^

How does a dysregulation of dopamine signaling and reward processing contribute to deficits in social behavior? There is a growing body of literature showing the brain reward circuitry is crucial in guiding social and nonsocial learning and behavior throughout development.^56^ Recent clinical information reports that individuals with autism have abnormal responses to rewards, leading to a new approach that considers autism from the perspective of reward processing deficits. Furthermore, the brain may respond to social sources of information in a manner similar to primary rewards, which are innately pleasurable. Research has shown that aberrant limbic mediation of the reward that drives social interaction may cause the social impairments seen in individuals with autism spectrum disorders.^58^ Thus, it has been suggested that the decreased feeling of pleasure during social exchanges could translate into a lack of interest in interacting with the social world.

The macro level alterations seen in brain activity and behavior in this study are likely caused by microscopic changes to dendritic spine morphology brought on by FXS,^57^ though proving a direct connection across these disparate levels of analysis will likely remain difficult for some time to come. The exact nature of the changes to dendritic spine morphology in FXS remains controversial.^59^ In addition, FXS produces differential effects in the different brain regions, particularly with regard to specific cortical layers.^59^ In the nucleus accumbens, medium spiny neurons experience NMDA receptor-dependent long-term potentiation that is completely absent in *fmr1*-KO mice.^57^ Furthermore, the nucleus accumbens core in *fmr1*-KO mice is also marked by elongated spines and an increase in filopodial spines on medium spiny neuron spines.^57^

Human fMRI studies have identified neural substrates involved in social recognition and the emotional valence assigned to familiar and unfamiliar faces.^60, 61, 62^ The discrimination between family members and strangers is highly relevant in the context of procreation and evolution.^63^ In ASD, the inability to socially engage or lack of social motivation has been attributed to a decreased reward value for social stimuli. Although people with autism show a similar activation to familial faces in the fusiform face area as compared with healthy controls, they show reduced functional connectivity from the fusiform face area to other brain areas like the amygdala,^64^ suggesting a disconnection with assigning the appropriate emotional valence to the face. Furthermore, while subjects with ASD have the ability to process familiar faces they show reduced activation to the faces of strangers again suggesting dysfunctional socioemotional processing.^64^ Eye-gaze aversion is a hallmark of both ASD and FXS. Using fMRI, it was found that subjects with FXS do not habituate but sensitize to face/eye gaze.^65^

Although FXS and ASD are different disorders, our finding that odor processing is altered in FX rats may have a human parallel, as Rozenkrantz *et al.*^66^ found children with ASD do not distinguish between pleasant and unpleasant odors as determined by the level of sensory-motor coordination involved in sniffing. The authors hold to the idea that the brain has hard-wired templates called internal action models^67^ for sensory-motor coordination in the area of social chemosignalling. Altering these internal action models makes the connection between ASD and dysregulation in olfaction.

### Caveats

For any imaging study on awake animals, the issues and consequences related to the stress of head restraint and restricted body movement must be considered. Protocols have been developed to help lessen the stress of an imaging study by acclimating animals to the environment of the MR scanner and the restraining devices helping to reduce stress hormones levels and measures of sympathetic autonomic activity.^34, 68^ These acclimation procedures put animals through several simulated imaging sessions and have been used to study sexual arousal in monkeys,^69^ generalized seizures in rats and monkeys^70, 71^ and exposure to psychostimulants like cocaine,^72, 73, 74^ nicotine,^75^ amphetamine^76^ and apomorphine.^68, 77^ Nonetheless, one must consider the experimental confound that exists with low levels of arousal and stress associated with imaging awake animals. Indeed, there is evidence showing a dysregulation in the hypothalamic–pituitary–adrenal axis in FX mice as compared with WT in response to a single exposure to immobilization stress.^78, 79^

One consideration when interpreting the data is the morphological changes in the brain structure that may occur between WT and FX rats. Ellegood *et al.*^16^ performed high-resolution MRI *Fmr1* KO mice and could only find a decrease in brain volume in the vermis of the cerebellum, specifically the deep cerebellar fastigial and interposed nuclei as compared with WT. In the present study, we analyzed the volumes from 171 brain areas using the 3D segmented rat atlas and found only five areas that were significantly different between WT and FX rats: cochlear nucleus, frontal association cortex, ventral medial striatum, premammillary nucleus and ventral orbital cortex ventral (See Supplementary Table 3). The general trend across the entire brain was a modest but insignificant increase in volume across all the areas. Hence, the possibility exists that regional differences in brain volumes in the FX rats may have altered the BOLD signal analysis particularly when the data are reported as volume of activation, that is, number of voxels activated in a 3D brain volume. To control for this possibility, we normalized the volume of activation to the brain volume of interest for each subject before statistical comparisons for both the genotypes.

### Summary

The prospective capability of animal imaging to follow changes in brain neurobiology following genetic or environmental insult has great value in the field of autism as one can follow the etiology and pathophysiology of disease progression. In addition, the combination of awake fMRI in rats with an imaging genetics approach^80^ represents a powerful experimental strategy that permits the identification of the effect of single gene mutations on neural circuits regulating emotion and cognition. When this neural activity is combined with a 3D segmented and annotated MRI rat atlas, it is possible to reconstruct distributed integrated neural circuits both in 3D and 2D that ‘finger print' the pattern of brain activity to a provocation paradigm. In this case here, we challenged FX rats to odor of almond associated with reward. Although it is only speculative, we would suggest that the perception of this odorant signal has been conserved over evolution and, in addition, assigned a high level of emotional valence as seen in the limbic circuit of Papez. The integration of these critical environment signals into the basal ganglia to regulate approach/avoidance behavior has been disrupted by this single gene mutation. Although positive BOLD response to almond odor was mostly similar to WT in the primary olfactory system, FX rats displayed a high degree of both positive as well as negative BOLD signal in the mesolimbic/habenular reward circuit, which may have contributed to the lack of a behavioral preference for such odor. The results of the present study support the growing body of evidence that reward processing is disrupted in FXS.
