# Supplementary material for: Functional magnetic resonance imaging in awake transgenic fragile X rats: evidence of dysregulation in reward processing in the mesolimbic/habenular neural circuit
Source: Transl Psychiatry. 2016 Mar 22;6(3):e763–. doi: 10.1038/tp.2016.15 (PMC4872441; doi:10.1038/tp.2016.15)
Supplement: Supplementary Table 1 [file tp201615x1.pdf]

Volume of Activation, Positive BOLD

Region of Interest(ROI)	WT ambient air			WT almond			*Fmr1* -KO almond			
	Med	Max	Min	Med	Max	Min	Med	Max	Min	P val
1st cerebellar lobule	0	14	0	0	22	0	15	33	6	0
perirhinal ctx	2	6	0	10	19	0	9	16	1	0.001
lemniscal nucleus	2	11	0	14	27	5	8	23	0	0.002
lateral septal nucleus	2	13	0	6	21	1	15	21	2	0.002
diagonal band of Broca	0	14	0	13	40	0	15	64	0	0.002
interpeduncular nucleus	0	14	0	6	24	0	13	57	0	0.002
reticular nucleus	0	4	0	2	23	0	6	20	0	0.002
2nd cerebellar lobule	3	7	0	7	17	1	10	40	1	0.002
central medial thalamic nucleus	0	0	0	13	58	0	6	38	0	0.003
insular ctx	1	3	0	3	26	1	5	13	0	0.004
caudal piriform ctx	1	5	0	4	45	0	4	11	1	0.005
globus pallidus	0	0	0	3	6	0	5	35	0	0.005
olfactory tubercles	2	11	0	10	39	4	11	20	0	0.005
lateral hypothalamus	3	10	0	10	17	2	14	25	0	0.006
infralimbic ctx	1	15	0	11	24	3	7	18	0	0.006
supramammillary nucleus	0	44	0	25	57	0	4	33	0	0.007
reuniens nucleus	0	5	0	0	17	0	7	22	0	0.007
ventromedial thalamic nucleus	0	0	0	0	9	0	5	11	0	0.007
primary somatosensory ctx forelimb	0	4	0	2	23	0	6	25	0	0.008
prelimbic ctx	1	7	0	6	27	1	3	24	0	0.008
secondary motor ctx	4	15	0	15	25	5	8	19	1	0.008
lateral orbital ctx	1	2	0	4	21	0	5	22	1	0.008
habenula nucleus	9	26	0	18	39	10	13	35	0	0.009
CA3 dorsal hippocampus	1	6	0	6	23	1	7	23	0	0.01
ventral medial striatum	0	2	0	1	10	0	4	18	0	0.011
arcuate nucleus hypothalamus	0	11	0	0	50	0	13	50	0	0.012
zona incerta	0	3	0	4	8	0	12	19	0	0.012
dentate gyrus dorsal	3	10	0	6	25	2	12	26	1	0.012
medial amygdaloid nucleus	1	16	0	15	32	0	11	24	0	0.013
anterior olfactory nucleus	1	5	0	4	31	1	4	15	1	0.013
lateral geniculate	1	18	0	4	16	0	16	30	0	0.014
simple lobule cerebellum	4	10	0	9	21	3	12	24	2	0.014
primary somatosensory ctx barrel field	1	5	0	3	27	1	5	18	0	0.014
dorsal lateral striatum	0	3	0	0	13	0	4	14	0	0.015
accumbens shell	0	6	0	3	15	1	6	13	0	0.015
medial dorsal thalamic nucleus	0	9	0	9	32	3	7	25	0	0.016
primary motor ctx	3	10	0	7	19	1	8	26	0	0.018
auditory ctx	1	2	0	2	16	0	4	13	0	0.018
tenia tecta ctx	5	14	0	21	51	3	8	42	0	0.019
visual 2 ctx	1	10	0	3	13	0	5	14	0	0.019
CA1 dorsal hippocampus	2	6	0	3	21	1	5	11	1	0.019
anterior amygdaloid nucleus	0	25	0	0	100	0	0	0	0	0.02
anterior cingulate area	2	14	0	11	37	1	7	23	1	0.021

5th cerebellar lobule	4	19	0	13	22	5	12	22	1	0.023
dorsal medial striatum	1	7	0	1	10	0	5	11	1	0.023
periaqueductal gray thalamus	3	10	0	9	21	3	11	22	1	0.023
CA3 ventral hippocampus	4	24	0	12	34	0	13	30	2	0.025
pontine nuclei	2	8	0	6	16	0	9	21	1	0.025
temporal ctx	0	4	0	1	11	0	6	13	0	0.026
paramedian lobule	0	14	0	4	15	0	7	30	1	0.026
secondary somatosensory ctx	1	4	0	2	16	0	3	9	0	0.028
cortical amygdaloid nucleus	1	6	0	10	17	0	4	13	0	0.028
inferior olivary complex	0	15	0	0	0	0	8	33	0	0.029
parabrachial nucleus	0	13	0	4	19	0	6	26	0	0.033
pedunculopontine tegmental area	0	13	0	6	17	0	6	23	0	0.033
paraventricular thalamic nucleus	9	29	0	26	32	0	23	50	4	0.033
ventral lateral striatum	1	3	0	1	13	0	5	15	0	0.033
intercalated amygdaloid nucleus	0	0	0	0	0	0	0	50	0	0.034
inferior colliculus	4	22	0	15	23	2	12	21	1	0.035
neural lobe pituitary	0	58	0	22	50	0	5	31	0	0.037
pontine reticular nucleus oral	0	4	0	1	10	0	3	15	0	0.038
4th cerebellar lobule	2	12	0	8	20	2	5	29	0	0.04
entorhinal ctx	4	7	0	10	15	1	8	19	1	0.04
flocculus cerebellum	1	12	0	13	27	0	9	16	0	0.042
extended amydala	0	18	0	0	17	0	10	40	0	0.043
primary somatosensory ctx jaw	0	4	0	1	21	0	3	24	0	0.045
posterior hypothalamic area	0	5	0	7	30	0	8	36	0	0.045
posterior thalamic nucleus	1	6	0	1	8	0	4	14	0	0.045
bed nucleus stria terminalis	1	7	0	2	17	0	8	22	0	0.048
rostral piriform ctx	2	10	0	5	26	2	5	11	0	0.049
primary somatosensory ctx shoulder	0	4	0	0	18	0	4	21	0	0.05
glomerular layer	4	20	0	17	35	0	13	28	0	0.051
supraoptic nucleus hypothalamus	0	0	0	0	100	0	0	100	0	0.052
central gray	4	16	0	4	33	0	13	22	0	0.053
accumbens core	0	2	0	1	8	0	2	7	0	0.053
ventrolateral thalamic nucleus	0	2	0	2	12	0	4	16	0	0.055
retrosplenial caudal ctx	1	13	0	7	36	0	9	27	0	0.056
3rd cerebellar lobule	3	13	0	11	25	1	8	22	1	0.057
paraventricular hypothalamic nucleus	0	22	0	17	67	0	16	50	0	0.059
ventral pallidum	2	6	0	3	14	0	9	18	0	0.061
magnocellular preoptic nucleus	0	60	0	17	83	0	10	56	0	0.067
ventral tegmental area	0	11	0	0	35	0	10	35	0	0.069
ectorhinal ctx	0	7	0	0	13	0	5	24	0	0.069
primary somatosensory ctx hindlimb	0	10	0	1	23	0	3	21	0	0.071
visual 1 ctx	2	14	0	3	8	0	6	16	0	0.072
crus 1 of ansiform lobule	4	9	0	8	21	3	8	24	2	0.073
lateral dorsal thalamic nucleus	0	50	0	9	33	0	13	33	0	0.074
substantia nigra compacta	0	30	0	0	42	0	12	30	0	0.074
root of trigeminal nerve	4	14	0	7	22	0	8	22	1	0.074
copula of the pyramis	0	50	0	6	19	1	8	44	2	0.08

parvicellular reticular nucleus	2	9	0	4	24	0	8	24	0	0.083
principal sensory nucleus trigeminal	1	9	0	5	9	0	7	25	0	0.085
ventral anterior thalamic nucleus	0	7	0	4	9	0	4	22	0	0.089
10th cerebellar lobule	0	19	0	7	16	0	15	33	0	0.092
sub coeruleus nucleus	1	4	0	4	21	0	4	20	0	0.092
parafascicular thalamic nucleus	1	8	0	4	17	0	8	21	0	0.093
lateral preoptic area	0	21	0	7	35	0	4	45	0	0.095
9th cerebellar lobule	0	100	0	1	7	0	6	32	0	0.098
motor trigeminal nucleus	2	4	0	0	29	0	9	14	0	0.099
retrosplenial rostral ctx	3	15	0	6	22	1	8	16	2	0.102
triangular septal nucleus	0	33	0	10	33	0	12	60	0	0.103
ventral medial nucleus	4	10	0	7	31	0	12	35	0	0.107
medial mammillary nucleus	0	36	0	26	50	0	10	33	0	0.107
facial nucleus	2	11	0	6	29	0	10	18	0	0.111
medial preoptic area	3	17	0	9	50	0	7	29	0	0.124
lateral posterior thalamic nucleus	4	20	0	9	27	0	8	24	2	0.131
paraflocculus cerebellum	5	14	0	11	23	0	9	21	3	0.144
central amygdaloid nucleus	2	6	0	4	26	0	11	16	0	0.144
8th cerebellar lobule	0	7	0	2	22	0	5	33	0	0.147
7th cerebellar lobule	0	50	0	0	21	0	6	34	0	0.154
parietal ctx	2	8	0	4	21	0	5	17	0	0.154
external plexiform layer	3	20	0	10	24	0	10	28	0	0.158
granular cell layer	2	15	0	6	17	0	12	21	0	0.159
primary somatosensory ctx upper lip	1	4	0	3	19	0	3	10	0	0.16
superior colliculus	4	24	0	9	21	1	9	22	5	0.161
medial geniculate	2	18	0	4	21	0	9	25	0	0.165
anterior thalamic nuclei	4	14	0	10	21	0	10	35	0	0.166
anterior hypothalamic area	4	11	0	7	21	0	17	25	0	0.19
ventral orbital ctx	1	5	0	2	16	0	3	17	0	0.194
anterior pretectal nucleus	0	22	0	9	19	0	7	26	0	0.199
reticular nucleus midbrain	3	20	0	3	6	0	5	13	0	0.2
medial cerebellar nucleus fastigial	0	17	0	0	21	0	0	30	0	0.206
substantia nigra reticularis	3	25	0	8	32	0	8	30	0	0.211
dorsal medial nucleus	0	0	0	0	50	0	0	100	0	0.247
dentate gyrus ventral	4	26	0	8	26	1	7	21	2	0.248
locus ceruleus	0	0	0	0	25	0	0	50	0	0.267
retrochiasmatic nucleus	0	25	0	0	67	0	0	0	0	0.27
claustrum	0	4	0	0	6	0	0	33	0	0.272
ventral posteriolmedial thalamic nucleus	0	3	0	0	4	0	1	10	0	0.275
dorsal paragigantocellularis nucleus	0	24	0	8	38	0	8	38	0	0.279
vestibular nucleus	3	16	0	4	16	0	9	27	0	0.287
dorsal raphe	0	22	0	0	50	0	0	22	0	0.31
subiculum dorsal	2	18	0	5	14	0	7	30	0	0.335
CA1 ventral hippocampus	2	9	0	4	18	0	5	25	0	0.336
interposed nucleus	0	10	0	0	7	0	4	13	0	0.346
medial septum	0	40	0	7	30	0	13	33	0	0.369
median raphe nucleus	0	19	0	0	9	0	2	24	0	0.381

gigantocellular reticular nucleus pons	2	11	0	3	9	0	4	23	0	0.382
endopiriform nucleus	0	4	0	0	16	0	2	17	0	0.394
ventral posteriolateral thalamic nucleus	1	2	0	1	10	0	3	15	0	0.398
reticulotegmental nucleus	0	25	0	0	13	0	0	0	0	0.409
medial orbital ctx	8	44	0	18	67	0	6	44	0	0.409
basal amygdaloid nucleus	2	7	0	3	16	1	4	16	0	0.476
cochlear nucleus	5	14	0	9	27	2	10	20	0	0.485
6th cerebellar lobule	5	27	0	7	28	1	12	25	1	0.491
lateral cerebellar nucleus	0	50	0	0	18	0	0	13	0	0.493
crus 2 of ansiform lobule	4	13	0	3	25	0	4	29	0	0.502
ventral subiculum	4	12	0	4	10	0	7	24	0	0.507
lateral amygdaloid nucleus	2	10	0	0	29	0	5	34	0	0.523
raphe linear	0	13	0	0	6	0	0	23	0	0.529
CA2 hippocampus	0	29	0	0	29	0	0	22	0	0.579
primary somatosensory ctx trunk	0	14	0	0	23	0	0	10	0	0.599
red nucleus	0	10	0	0	14	0	0	13	0	0.603
periolivary nucleus	2	20	0	5	20	0	4	21	0	0.613
substantia innominata	0	25	0	0	75	0	0	100	0	0.626
premammillary nucleus	0	29	0	0	57	0	0	25	0	0.657
raphe magnus	0	25	0	0	11	0	3	29	0	0.678
pineal gland	0	67	0	0	25	0	0	100	0	0.686
frontal association ctx	4	29	0	10	24	0	8	28	2	0.717
precuniform nucleus	0	9	0	0	10	0	0	8	0	0.757
anterior lobe pituitary	6	30	0	6	24	1	10	26	0	0.77
solitary tract nucleus	2	25	0	2	9	0	4	27	0	0.774
prerubral field	0	17	0	0	15	0	0	22	0	0.803
trapezoid body	0	19	0	4	19	0	2	28	0	0.897
dorsomedial tegmental area	2	13	0	3	24	0	3	23	0	0.94
pontine reticular nucleus caudal	2	9	0	2	10	1	2	21	0	0.952
subthalamic nucleus	0	50	0	0	33	0	0	50	0	0.988
suprachiasmatic nucleus	0	0	0	0	0	0	0	0	0	NaN
