# Supplementary material for: Functional magnetic resonance imaging in awake transgenic fragile X rats: evidence of dysregulation in reward processing in the mesolimbic/habenular neural circuit
Source: Transl Psychiatry. 2016 Mar 22;6(3):e763–. doi: 10.1038/tp.2016.15 (PMC4872441; doi:10.1038/tp.2016.15)
Supplement: Supplementary Table 2 [file tp201615x2.pdf]

Volume of Activation, Negative BOLD

Region of Interest(ROI)	WT ambiet air			WT almond			*Fmr1* -KO almond			
	Med	Max	Min	Med	Max	Min	Med	Max	Min	P val
visual 2 ctx	0	4	0	2	5	0	6	12	0	0.001
3rd cerebellar lobule	2	8	0	8	32	0	15	35	3	0.001
CA1 dorsal hippocampus	0	3	0	2	7	0	5	20	1	0.002
retrosplenial rostral ctx	3	9	0	6	12	3	14	32	3	0.002
lateral geniculate	0	10	0	4	20	0	14	30	0	0.003
primary somatosensory ctx shoulder	0	0	0	0	16	0	4	19	0	0.003
secondary motor ctx	3	13	0	12	18	3	15	28	1	0.003
crus 2 of ansiform lobule	0	6	0	8	45	0	10	26	0	0.004
crus 1 of ansiform lobule	2	9	0	10	43	2	14	27	1	0.004
7th cerebellar lobule	0	0	0	0	39	0	5	44	0	0.004
primary somatosensory ctx trunk	0	5	0	0	6	0	3	13	0	0.004
inferior olivary complex	0	23	0	0	0	0	5	27	0	0.004
reticular nucleus	0	4	0	2	6	0	10	30	0	0.005
primary somatosensory ctx hindlimb	1	3	0	1	3	0	4	11	0	0.005
primary motor ctx	2	6	0	5	12	2	12	23	1	0.005
primary somatosensory ctx jaw	0	4	0	1	10	0	6	20	0	0.005
5th cerebellar lobule	5	15	1	12	29	2	16	38	4	0.005
entorhinal ctx	2	10	0	4	19	1	11	21	2	0.005
ventral subiculum	1	9	0	5	20	0	11	20	0	0.006
visual 1 ctx	1	5	0	1	6	0	3	11	1	0.006
4th cerebellar lobule	2	9	0	7	28	0	15	30	2	0.006
2nd cerebellar lobule	1	6	0	4	36	0	11	37	1	0.006
temporal ctx	0	14	0	1	6	0	7	13	0	0.006
medial geniculate	0	9	0	8	22	0	14	41	0	0.006
dentate gyrus dorsal	1	5	0	6	16	0	11	25	0	0.007
superior colliculus	2	14	0	10	18	0	14	25	1	0.007
periaqueductal gray thalamus	2	5	0	7	13	0	13	26	0	0.008
copula of the pyramis	0	7	0	5	33	0	11	38	0	0.009
auditory ctx	0	4	0	1	4	0	3	10	1	0.009
retrosplenial caudal ctx	2	13	0	5	13	1	12	28	0	0.009
anterior cingulate area	1	9	0	5	11	2	6	28	1	0.01
substantia nigra compacta	0	6	0	0	17	0	8	31	0	0.011
rostral piriform ctx	1	5	0	7	19	0	14	22	0	0.012
lateral orbital ctx	1	2	0	2	18	0	3	14	1	0.012
10th cerebellar lobule	0	28	0	7	56	0	6	30	0	0.013
paraflocculus cerebellum	2	16	0	11	45	1	16	26	1	0.013
anterior hypothalamic area	1	12	0	7	17	0	17	42	0	0.013
accumbens core	0	2	0	0	2	0	4	6	0	0.013
lateral septal nucleus	3	8	0	4	19	0	11	40	2	0.013
root of trigeminal nerve	3	12	0	7	31	1	15	36	1	0.015
granular cell layer	1	8	0	4	13	0	13	30	0	0.016
lateral preoptic area	0	17	0	0	14	0	12	26	0	0.016
insular ctx	1	3	0	4	14	0	7	19	0	0.017

caudal piriform ctx	1	4	0	4	21	0	9	29	0	0.017
anterior thalamic nuclei	2	9	0	8	28	0	10	31	0	0.018
zona incerta	1	4	0	4	8	0	7	15	0	0.02
reticular nucleus midbrain	1	4	0	5	8	0	7	16	0	0.02
cortical amygdaloid nucleus	0	6	0	7	21	0	8	24	0	0.021
lateral dorsal thalamic nucleus	0	19	0	0	33	0	17	70	0	0.021
supramammillary nucleus	0	0	0	0	20	0	4	44	0	0.023
ventral tegmental area	0	0	0	0	20	0	3	37	0	0.023
ventral pallidum	1	6	0	3	8	0	9	18	0	0.024
CA3 dorsal hippocampus	1	9	0	3	15	1	9	27	0	0.024
pontine nuclei	1	10	0	2	26	0	6	18	0	0.024
ventral posteriolateral thalamic nucleus	0	10	0	0	5	0	6	14	0	0.027
primary somatosensory ctx forelimb	1	2	0	1	9	0	4	22	0	0.028
paramedian lobule	1	9	0	4	45	0	7	20	0	0.028
central amygdaloid nucleus	1	10	0	4	16	0	9	30	0	0.029
primary somatosensory ctx upper lip	0	4	0	1	2	0	3	13	0	0.031
perirhinal ctx	2	21	0	7	16	0	12	19	3	0.031
inferior colliculus	3	22	0	14	21	0	13	30	2	0.032
substantia innominata	0	0	0	0	0	0	0	50	0	0.035
median raphe nucleus	0	0	0	0	17	0	2	9	0	0.035
lemniscal nucleus	2	18	0	6	37	0	13	25	0	0.036
medial cerebellar nucleus fastigial	0	13	0	6	37	0	8	44	0	0.037
external plexiform layer	2	7	0	4	22	0	17	40	0	0.039
interposed nucleus	0	20	0	8	41	0	5	30	0	0.039
prelimbic ctx	1	5	0	4	15	0	4	18	1	0.041
olfactory tubercles	2	10	0	4	17	1	17	31	0	0.041
bed nucleus stria terminalis	0	3	0	1	18	0	3	21	0	0.043
paraventricular thalamic nucleus	0	20	0	16	39	0	16	44	0	0.044
principal sensory nucleus trigeminal	3	20	0	6	34	0	11	23	2	0.044
dentate gyrus ventral	2	24	0	7	17	0	9	33	0	0.044
simple lobule cerebellum	5	18	1	10	33	0	19	28	3	0.044
posterior hypothalamic area	0	11	0	4	31	0	10	33	0	0.044
lateral hypothalamus	2	6	0	6	17	1	13	30	0	0.045
anterior olfactory nucleus	1	6	0	4	18	0	13	27	0	0.046
infralimbic ctx	1	19	0	8	14	0	6	25	1	0.049
ventrolateral thalamic nucleus	0	5	0	1	2	0	3	26	0	0.049
secondary somatosensory ctx	0	2	0	2	2	0	2	10	0	0.05
dorsal raphe	0	29	0	0	30	0	13	67	0	0.052
glomerular layer	3	18	0	9	26	0	14	41	0	0.055
dorsal lateral striatum	0	1	0	0	6	0	2	6	0	0.058
parabrachial nucleus	0	12	0	2	29	0	9	28	0	0.058
ventral medial striatum	0	2	0	1	4	0	2	14	0	0.061
substantia nigra reticularis	3	20	0	6	23	0	13	27	0	0.064
red nucleus	0	15	0	0	7	0	4	25	0	0.065
dorsal medial striatum	1	6	0	1	9	0	5	15	0	0.066
central gray	5	13	0	2	30	0	16	30	0	0.068
interpeduncular nucleus	0	14	0	3	29	0	7	21	0	0.069

ventromedial thalamic nucleus	0	2	0	2	4	0	3	15	0	0.07
9th cerebellar lobule	0	5	0	3	42	0	5	22	0	0.072
frontal association ctx	4	33	0	13	37	0	22	51	0	0.072
parvicellular reticular nucleus	1	29	0	2	38	1	9	28	0	0.072
pontine reticular nucleus oral	0	6	0	1	11	0	2	6	0	0.078
flocculus cerebellum	2	24	0	9	33	2	9	29	2	0.079
primary somatosensory ctx barrel field	1	6	0	3	4	0	4	22	0	0.081
ventral posteriolmedial thalamic nucleus	0	3	0	1	7	0	2	12	0	0.085
medial dorsal thalamic nucleus	0	14	0	6	26	0	9	22	0	0.086
locus ceruleus	0	25	0	0	33	0	0	100	0	0.087
lateral cerebellar nucleus	0	14	0	0	20	0	5	56	0	0.091
6th cerebellar lobule	5	25	1	13	37	2	18	40	1	0.093
magnocellular preoptic nucleus	0	17	0	11	33	0	24	40	0	0.094
dorsal paragigantocellularis nucleus	0	47	0	8	54	0	13	42	0	0.096
periolivary nucleus	3	11	0	13	30	0	7	22	0	0.1
habenula nucleus	8	17	0	7	31	0	17	55	2	0.1
CA1 ventral hippocampus	0	11	0	1	32	0	5	21	0	0.101
ectorhinal ctx	0	25	0	3	8	0	4	18	0	0.104
CA3 ventral hippocampus	4	12	0	11	32	0	9	32	0	0.104
solitary tract nucleus	0	25	0	9	39	0	3	40	0	0.111
ventral lateral striatum	1	2	0	0	6	0	2	7	0	0.112
diagonal band of Broca	0	24	0	4	47	0	15	33	0	0.113
gigantocellular reticular nucleus pons	0	30	0	5	45	0	6	30	0	0.12
medial preoptic area	3	21	0	8	23	0	14	35	0	0.12
lateral posterior thalamic nucleus	3	19	0	3	18	0	18	46	0	0.121
medial orbital ctx	0	16	0	10	44	0	14	42	0	0.122
endopiriform nucleus	0	4	0	1	7	0	4	9	0	0.125
medial mammillary nucleus	0	14	0	4	50	0	7	33	0	0.127
ventral anterior thalamic nucleus	0	13	0	0	12	0	4	19	0	0.133
neural lobe pituitary	0	33	0	0	50	0	7	64	0	0.134
subiculum dorsal	2	8	0	2	13	0	8	24	0	0.149
ventral medial nucleus	4	26	0	4	31	0	18	32	0	0.157
posterior thalamic nucleus	0	7	0	2	9	0	3	12	0	0.157
anterior pretectal nucleus	0	13	0	4	13	0	14	31	0	0.158
central medial thalamic nucleus	0	27	0	0	13	0	7	33	0	0.165
vestibular nucleus	3	14	0	6	49	0	12	30	1	0.166
parietal ctx	1	9	0	2	7	0	5	16	0	0.173
reuniens nucleus	0	16	0	0	20	0	6	18	0	0.173
8th cerebellar lobule	0	10	0	0	27	0	3	23	0	0.175
anterior lobe pituitary	1	12	0	1	30	0	14	26	0	0.179
tenia tecta ctx	3	18	0	7	21	0	17	34	0	0.181
cochlear nucleus	7	18	0	10	45	0	10	23	0	0.189
medial septum	0	36	0	0	31	0	6	38	0	0.19
accumbens shell	1	4	0	2	7	0	9	20	0	0.196
globus pallidus	0	3	0	0	5	0	0	8	0	0.204
pedunculopontine tegmental area	0	9	0	0	9	0	0	24	0	0.216
raphe linear	0	21	0	0	10	0	0	9	0	0.227

medial amygdaloid nucleus	2	22	0	2	25	0	10	37	0	0.239
dorsomedial tegmental area	6	21	0	0	7	0	2	16	0	0.249
intercalated amygdaloid nucleus	0	0	0	0	25	0	0	33	0	0.286
anterior amygdaloid nucleus	0	0	0	0	17	0	0	0	0	0.295
dorsal medial nucleus	0	0	0	0	20	0	0	0	0	0.295
pineal gland	0	67	0	0	50	0	0	33	0	0.301
triangular septal nucleus	0	29	0	7	50	0	9	23	0	0.321
paraventricular hypothalamic nucleus	0	29	0	0	20	0	0	43	0	0.362
parafascicular thalamic nucleus	1	9	0	1	6	0	3	16	0	0.411
basal amygdaloid nucleus	2	4	0	5	15	0	3	18	0	0.422
sub coeruleus nucleus	0	17	0	4	20	0	4	20	0	0.444
motor trigeminal nucleus	0	8	0	0	29	0	2	24	0	0.5
facial nucleus	4	14	0	4	32	0	5	33	0	0.507
supraoptic nucleus hypothalamus	0	50	0	0	100	0	0	50	0	0.623
retrochiasmatic nucleus	0	50	0	0	0	0	0	67	0	0.641
lateral amygdaloid nucleus	0	17	0	3	32	0	2	23	0	0.664
precuniform nucleus	0	100	0	0	15	0	0	50	0	0.691
ventral orbital ctx	0	5	0	1	10	0	1	5	0	0.707
prerubral field	0	15	0	0	36	0	0	20	0	0.759
1st cerebellar lobule	0	30	0	0	46	0	7	58	0	0.77
reticulotegmental nucleus	0	14	0	0	33	0	0	13	0	0.777
arcuate nucleus hypothalamus	0	33	0	0	50	0	0	44	0	0.821
extended amydala	0	27	0	0	17	0	0	20	0	0.84
subthalamic nucleus	0	100	0	0	33	0	0	100	0	0.888
CA2 hippocampus	0	13	0	0	14	0	0	38	0	0.895
trapezoid body	2	33	0	0	23	0	2	14	0	0.946
raphe magnus	4	28	0	5	44	0	5	25	0	0.962
pontine reticular nucleus caudal	3	18	0	2	11	0	3	12	0	0.967
claustrum	0	10	0	0	12	0	0	8	0	0.993
premammillary nucleus	0	75	0	0	50	0	0	67	0	0.997
suprachiasmatic nucleus	0	0	0	0	0	0	0	0	0	NaN
