# Supplementary material for: Functional magnetic resonance imaging in awake transgenic fragile X rats: evidence of dysregulation in reward processing in the mesolimbic/habenular neural circuit
Source: Transl Psychiatry. 2016 Mar 22;6(3):e763–. doi: 10.1038/tp.2016.15 (PMC4872441; doi:10.1038/tp.2016.15)
Supplement: Supplementary Table 3 [file tp201615x3.pdf]

	Wild-Type		Fmr1 KO		
Region of Interest(ROI)	Vol mm^3	SE	Vol mm^3	SE	P val
cochlear nucleus	3.99	0.17	4.75	0.18	0.006
frontal association ctx	11.02	0.34	13.51	0.91	0.014
ventral medial striatum	8.92	0.32	9.88	0.20	0.022
premammillary nucleus	0.84	0.08	1.08	0.07	0.044
ventral orbital ctx	5.27	0.25	6.21	0.39	0.048
neural lobe pituitary	0.86	0.06	1.05	0.07	0.059
Ventricle	6.04	0.28	6.85	0.32	0.066
ventral medial nucleus	1.52	0.08	1.71	0.06	0.084
infralimbic ctx	7.17	0.49	8.29	0.39	0.090
medial mammillary nucleus	1.51	0.11	1.74	0.06	0.094
arcuate nucleus	0.82	0.06	0.98	0.06	0.095
lateral posterior thalamic nucleus	6.31	0.39	7.15	0.29	0.102
pontine reticular nucleus oral	7.69	0.54	9.12	0.66	0.105
parabrachial nucleus	4.80	0.24	5.34	0.22	0.112
paraflocculus cerebellum	26.98	1.12	29.40	0.93	0.114
endopiriform nucleus	12.82	0.42	13.65	0.26	0.116
cortical amygdaloid nucleus	7.29	0.26	7.81	0.19	0.130
medial septum	1.25	0.07	1.41	0.08	0.134
basal amygdaloid nucleus	13.37	0.41	14.22	0.37	0.140
dentate gyrus dorsal	15.05	0.62	16.11	0.24	0.142
DPGi	2.49	0.21	2.94	0.22	0.149
simple lobule cerebellum	17.09	1.09	19.46	1.22	0.157
CA1 dorsal	25.07	0.89	26.52	0.33	0.159
medial preoptic area	3.09	0.19	3.46	0.19	0.172
anterior olfactory nucleus	16.79	0.63	17.80	0.27	0.172
medial cerebellar nucleus fastigial	1.18	0.13	1.44	0.13	0.182
pontine nuclei	10.73	0.56	11.70	0.40	0.184
lateral septal nucleus	19.17	1.07	20.89	0.57	0.184
claustrum	10.63	0.19	11.05	0.25	0.184
retrosplenial caudal ctx	30.60	1.31	32.84	0.91	0.185
solitary tract nucleus	2.72	0.29	3.28	0.33	0.212
White Matter	11.02	0.69	12.32	0.77	0.214
crus 1 of ansiform lobule	24.50	2.63	28.44	1.61	0.228
olfactory tubercles	14.71	0.61	15.67	0.47	0.232
lemniscal nucleus	5.42	0.36	6.09	0.42	0.233
dorsal medial striatum	20.41	1.20	22.20	0.79	0.235
secondary motor ctx	22.82	0.72	23.87	0.44	0.235
ventral tegmental area	1.42	0.18	1.73	0.19	0.239
entorhinal ctx	43.59	1.90	46.49	1.41	0.241
prelimbic ctx	11.75	0.38	12.39	0.40	0.247
dorsal lateral striatum	33.91	0.91	35.35	0.79	0.247
lateral orbital ctx	13.51	0.54	14.31	0.38	0.248

central medial thalamic nucleus	2.28	0.07	2.15	0.09	0.250
prerubral field	1.99	0.12	1.75	0.16	0.252
perirhinal ctx	13.62	0.57	14.53	0.51	0.252
lateral geniculate	5.28	0.30	5.69	0.15	0.255
caudal piriform ctx	29.11	1.10	30.63	0.63	0.257
root of trigeminal nerve	14.53	0.66	15.65	0.73	0.263
medial amygdaloid nucleus	3.70	0.25	4.09	0.24	0.267
anterior hypothalamic area	6.48	0.37	5.99	0.19	0.268
facial nucleus	6.74	0.32	7.26	0.33	0.271
median raphe nucleus	2.25	0.19	2.58	0.23	0.276
paraventricular nucleus	1.66	0.12	1.91	0.20	0.277
bed nucleus stria terminalis	4.64	0.17	4.91	0.17	0.278
magnocellular preoptic nucleus	1.62	0.12	1.79	0.09	0.279
reticular nucleus midbrain	20.54	0.98	21.96	0.84	0.285
insular ctx	58.68	1.78	60.94	1.03	0.297
trapezoid body	2.31	0.17	2.55	0.15	0.298
visual 1 ctx	25.80	1.11	27.35	0.94	0.301
ventral posteriolateral thalamic nucleu	5.06	0.23	5.43	0.27	0.303
ventral anterior thalamic nucleus	2.59	0.31	3.04	0.29	0.307
red nucleus	1.75	0.08	1.88	0.10	0.325
diagonal band of Broca	3.63	0.21	3.92	0.18	0.327
CA1 hippocampus ventral	11.90	0.44	12.44	0.28	0.332
lateral preoptic area	3.00	0.18	3.25	0.17	0.341
anterior lobe pituitary	6.77	0.49	7.37	0.35	0.342
supraoptic nucleus	0.63	0.05	0.53	0.10	0.343
entral posteriolmedial thalamic nucleu	5.24	0.34	5.67	0.30	0.344
locus ceruleus	0.82	0.08	0.91	0.06	0.352
triangular septal nucleus	0.60	0.19	0.92	0.30	0.356
superior colliculus	20.12	1.23	21.60	0.97	0.359
substantia nigra reticularis	5.18	0.43	5.68	0.37	0.388
interposed nucleus	2.07	0.15	2.27	0.18	0.390
PCRt	13.21	1.09	14.66	1.32	0.394
pineal gland	0.46	0.04	0.50	0.04	0.404
anterior thalamic nuclei	8.83	0.35	8.46	0.26	0.408
substantia nigra compacta	1.90	0.12	2.07	0.16	0.411
tenia tecta ctx	5.64	0.30	5.93	0.16	0.412
substantia innominata	0.45	0.07	0.54	0.08	0.414
vestibular nucleus	9.56	0.48	10.13	0.50	0.415
medial pretectal area	0.88	0.14	1.04	0.13	0.418
White Matter	98.56	3.05	101.59	1.85	0.418
reticulotegmental nucleus	0.78	0.10	0.91	0.13	0.418
ectorhinal ctx	4.26	0.41	4.76	0.47	0.420
6th cerebellar lobule	10.12	1.73	11.92	1.34	0.425
auditory ctx	35.58	1.07	36.65	0.72	0.426

visual 2 ctx	25.70	0.95	26.62	0.58	0.428
medial orbital ctx	3.50	0.33	3.15	0.27	0.428
principal sensory nucleus trigeminal	9.07	0.61	9.75	0.60	0.431
3rd cerebellar lobule	20.57	0.77	21.49	0.88	0.431
supramammillary nucleus	0.87	0.06	0.80	0.07	0.435
lateral dorsal thalamic nucleus	1.71	0.15	1.54	0.16	0.438
suprachiasmatic nucleus	0.31	0.05	0.37	0.05	0.444
paraventricular nuclus	0.80	0.05	0.75	0.04	0.459
primary somatosensory ctx shoulder	3.18	0.24	3.43	0.28	0.490
extended amydala	4.07	0.26	3.86	0.14	0.490
2nd cerebellar lobule	17.21	0.75	17.82	0.38	0.492
9th cerebellar lobule	2.67	0.65	3.23	0.45	0.493
posterior thalamic nucleus	11.84	0.46	12.29	0.50	0.501
1st cerebellar lobule	2.64	0.15	2.78	0.15	0.514
accumbens core	11.15	0.54	11.59	0.38	0.520
ventral lateral striatum	28.93	0.83	29.59	0.53	0.522
interpeduncular nucleus	2.34	0.20	2.53	0.23	0.528
accumbens shell	12.80	0.48	13.18	0.31	0.528
CA2	2.35	0.14	2.46	0.11	0.528
primary somatosensory ctx jaw	27.46	0.68	28.33	1.25	0.529
paramedian lobule	4.40	1.11	5.36	1.03	0.532
granular cell layer	20.39	1.28	21.67	1.72	0.547
10th cerebellar lobule	2.29	0.43	2.64	0.38	0.547
glomerular layer	18.17	1.02	19.10	1.16	0.547
lateral amygdaloid nucleus	2.72	0.09	2.81	0.11	0.548
intercalated amygdaloid nucleus	0.50	0.05	0.54	0.06	0.558
globus pallidus	8.96	0.34	8.72	0.21	0.561
central gray	4.39	0.28	4.59	0.21	0.569
ventromedial thalamic nucleus	3.80	0.22	3.96	0.18	0.571
periolivary nucleus	4.01	0.16	3.88	0.15	0.576
raphe linear	1.78	0.14	1.89	0.12	0.576
raphe magnus	2.03	0.08	2.09	0.08	0.583
retrosplenial rostral ctx	11.31	0.61	10.85	0.66	0.606
pontine reticular nucleus caudal	14.91	0.90	15.57	0.90	0.607
zona incerta	5.33	0.20	5.46	0.15	0.607
subiculum dorsal	9.84	0.50	10.29	0.78	0.621
temporal ctx	6.63	0.44	6.96	0.52	0.627
flocculus cerebellum	8.82	0.80	9.29	0.48	0.630
primary somatosensory ctx barrel field	38.28	1.66	39.25	0.97	0.630
dentate gyrus ventral	9.75	0.46	10.10	0.62	0.638
reticular nucleus	10.60	0.63	10.27	0.25	0.645
parafascicular thalamic nucleus	7.58	0.25	7.73	0.20	0.647
lateral cerebellar nucleus	1.47	0.10	1.55	0.17	0.647
anterior cingulate area	32.74	1.18	32.08	0.78	0.652

dorsal medial nucleus	1.45	0.09	1.49	0.04	0.652
inferior olivary complex	1.49	0.30	1.69	0.35	0.665
raphe obscurus nucleus	0.42	0.10	0.48	0.14	0.706
dorsomedial tegmental area	2.97	0.24	3.09	0.17	0.709
CA3 dorsal	16.90	0.78	17.22	0.25	0.712
ventral subiculum	11.13	0.73	10.63	1.19	0.714
gigantocellular reticular nucleus pons	16.37	1.33	17.21	2.04	0.723
primary somatosensory ctx trunk	4.22	0.44	3.97	0.58	0.728
periaqueductal gray thalamus	17.58	0.54	17.80	0.35	0.735
8th cerebellar lobule	2.17	0.58	2.43	0.53	0.745
primary somatosensory ctx forelimb	20.98	1.07	21.41	0.84	0.757
rostral piriform ctx	28.30	0.80	28.73	1.21	0.763
primary motor ctx	51.68	1.50	52.28	1.26	0.764
habenula nucleus	3.42	0.18	3.49	0.16	0.784
CA3 hippocampus ventral	8.19	0.47	8.03	0.40	0.788
copula of the pyramis	3.15	0.76	3.42	0.65	0.789
5th cerebellar lobule	30.48	1.15	29.96	1.63	0.794
pedunculopontine tegmental area	2.30	0.27	2.42	0.38	0.802
dorsal raphe	1.42	0.15	1.48	0.19	0.811
sub coeruleus nucleus	4.77	0.34	4.67	0.41	0.848
parietal ctx	14.36	0.56	14.48	0.42	0.862
precuniform nucleus	2.46	0.27	2.38	0.33	0.864
ventrolateral thalamic nucleus	5.64	0.64	5.46	0.87	0.864
primary somatosensory ctx upper lip	25.53	0.87	25.32	1.17	0.887
medial geniculate	4.88	0.23	4.83	0.30	0.891
anterior pretectal nucleus	5.56	0.43	5.64	0.48	0.895
medial dorsal thalamic nucleus	3.91	0.15	3.86	0.36	0.897
secondary somaotsensory ctx	18.00	0.77	18.12	0.60	0.905
central amygdaloid nucleus	6.32	0.26	6.36	0.16	0.906
4th cerebellar lobule	18.35	0.65	18.47	0.81	0.906
olivary nucleus	1.08	0.12	1.09	0.14	0.918
inferior colliculus	24.17	0.92	24.05	0.83	0.924
ventral pallidum	7.90	0.22	7.94	0.40	0.924
primary somatosensory ctx hindlimb	12.67	0.45	12.61	0.37	0.925
retrochiasmatic nucleus	0.71	0.09	0.70	0.07	0.943
crus 2 of ansiform lobule	4.10	1.22	3.98	1.12	0.945
anterior amygdaloid nucleus	3.69	0.47	3.65	0.47	0.954
lateral hypothalamus	21.58	0.59	21.53	0.45	0.954
external plexiform layer	17.40	1.19	17.30	1.26	0.955
subthalamic nucleus	0.98	0.08	0.97	0.08	0.958
reuniens nucleus	3.44	0.18	3.44	0.11	0.965
motor trigeminal nucleus	3.94	0.22	3.95	0.27	0.968
posterior hypothalamic area	3.58	0.14	3.57	0.18	0.974
7th cerebellar lobule	1.55	0.50	1.54	0.55	0.999
